# Subdiffusion Equation with Fractional Caputo Time Derivative with Respect to Another Function in Modeling Superdiffusion

**DOI:** 10.3390/e27010048

**Published:** 2025-01-09

**Authors:** Tadeusz Kosztołowicz

**Affiliations:** 1 Institute of Physics, Jan Kochanowski University, Uniwersytecka 7, 25-406 Kielce, Poland; tadeusz.kosztolowicz@ujk.edu.pl; 2 Department of Radiological Informatics and Statistics, Medical University of Gdańsk, Tuwima 15, 80-210 Gdańsk, Poland

**Keywords:** anomalous diffusion, *g*-superdiffusion, *g*-subdiffusion, fractional calculus, fractional Caputo derivative with respect to another function

## Abstract

Superdiffusion is usually defined as a random walk process of a molecule, in which the time evolution of the mean-squared displacement, $\sigma^{2}$, of the molecule is a power function of time, $\sigma^{2}\left(t\right)∼t^{2/\gamma }$, with γ∈(1,2). An equation with a Riesz-type fractional derivative of the order γ with respect to a spatial variable (a fractional superdiffusion equation) is often used to describe superdiffusion. However, this equation leads to the formula $\sigma^{2}\left(t\right)=\kappa t^{2/\gamma }$ with κ=∞, which, in practice, makes it impossible to define the parameter γ. Moreover, due to the nonlocal nature of this derivative, it is generally not possible to impose boundary conditions at a thin partially permeable membrane. We show a model of superdiffusion based on an equation in which there is a fractional Caputo time derivative with respect to another function, *g*; the spatial derivative is of the second order. By choosing the function in an appropriate way, we obtain the *g*-superdiffusion equation, in which Green’s function (GF) in the long time limit approaches GF for the fractional superdiffusion equation. GF for the *g*-superdiffusion equation generates $\sigma^{2}$ with finite κ. In addition, the boundary conditions at a thin membrane can be given in a similar way as for normal diffusion or subdiffusion. As an example, the filtration process generated by a partially permeable membrane in a superdiffusive medium is considered.

## 1. Introduction

Diffusion processes are generated by the random walks of molecules. In the standard continuous-time random walk (CTRW) model [1,2,3,4,5,6,7], this process is characterized by two probability densities, ψ(Δt) and λ(Δx), describing the jumps of a single particle; Δt is the waiting time for the particle to jump, and Δx is the length of the jump. In normal diffusion, both distributions have finite moments. In the case of anomalous diffusion, at least one of these distributions has a heavy tail, which causes it to have infinite moments. In the case of subdiffusion, the waiting time for the molecule to jump is anomalously long; thus, ψ has a heavy tail. Subdiffusion can occur in media in which the movement of molecules is very hindered, such as gels or porous media. When molecule jumps are anomalously long, which occurs in turbulent media, we are dealing with superdiffusion. In this case, λ has a heavy tail, so the second central moment of this distribution is infinite. Within the CTRW model, subdiffusion is described by an equation with a fractional time derivative, and the superdiffusion equation contains a fractional derivative with respect to the spatial variable [1,2,3,6,7,8,9,10,11,12,13,14,15].

Different types of diffusion processes are often defined by the temporal evolution of the mean-squared displacement (MSD), $\sigma^{2}$, of a diffusing molecule [1,16,17,18,19]:

(1) $$\begin{array}{c} \sigma^{2}\left(t\right)∼\left\{\begin{array}{c} t^{2/\gamma },\;2>\gamma >1,\;\mathrm{for}\;\mathrm{superdiffusion},\\ t,\;\mathrm{for}\;\mathrm{normal}\;\mathrm{diffusion},\\ t^{\alpha },\;0<\alpha <1,\;\mathrm{for}\;\mathrm{ordinary}\;\mathrm{subdiffusion}. \end{array}\right. \end{array}$$

In an unbounded homogeneous one-dimensional system, $\sigma^{2}\left(t\right)=2D_{\alpha }t^{\alpha }/\Gamma \left(1+\alpha \right)$, with 0<α<1 for subdiffusion and $\sigma^{2}\left(t\right)=2D_{\alpha =1}t$ for normal diffusion. $D_{\alpha }$ is a subdiffusion coefficient (or normal diffusion coefficient when α=1) given in units of ${m^{2}/\sec}^{\alpha }$. However, for superdiffusion described by the fractional superdiffusion equation, this relation takes the form

(2) $$\sigma^{2}\left(t\right)=\kappa t^{2/\gamma }$$

with κ=∞. Thus, for fractional superdiffusion, $\sigma^{2}\left(t\right)=\infty$ holds, which is a rather useless relation because it does not define the parameter γ.

Another disadvantage of the fractional superdiffusion model is the difficulty in assigning boundary conditions at a partially permeable membrane. The reason for this is that the fractional derivative with respect to the spatial variable has a nonlocal characteristic. So, nonlocal boundary conditions are used. This causes difficulties in using the fractional superdiffusion equation to model processes in membrane systems.

We propose a model of superdiffusion that leads to Equation (2) with κ<∞ and in which local boundary conditions, such as those for subdiffusion or normal diffusion, can be applied at a membrane. This model is based on the *g*-subdiffusion equation with a fractional Caputo time derivative with respect to another function, *g*; see Refs. [20,21]. The *g*-subdiffusion equation can be interpreted as the ordinary subdiffusion equation with a changed time variable, t→g(t). The time scale change in these diffusion equations was generated by a stochastic process within the subordinate method [6,22,23,24,25,26]. In our considerations, the change in the time variable is not related to the subordinate method. The *g*-subdiffusion equation has been used to describe a smooth transition from subdiffusion to ultraslow diffusion (slow subdiffusion) [20], superdiffusion [27], and subdiffusion with a changed α parameter [28]. In this paper, this equation is used to describe superdiffusion over the entire time domain. We consider diffusion in a one-dimensional unbounded system, except in Section 5, where the filtration process in a membrane system is modeled.

## 2. Anomalous Diffusion Equations

In this section, we show the ordinary subdiffusion and fractional superdiffusion equations, along with their Green’s functions. Green’s function (GF), $P(x,t|x_{0})$, is defined as the solution to an equation with the initial condition $P\left(x,0|x_{0}\right)=\delta \left(x-x_{0}\right)$, where δ is the delta-Dirac function, and, in an unbounded system, with the boundary conditions $P(\pm \infty ,t|x_{0})=0$. GF is interpreted as the probability density of finding a molecule at point *x* at time *t*, with $x_{0}$ being the initial position of the molecule at t=0.

### 2.1. Ordinary Subdiffusion Equation

The ordinary subdiffusion equation is

(3) ∂αCPα(x,t|x0)∂tα=Dα∂2Pα(x,t|x0)∂x2,

where 0<α<1,

(4) dαCf(t)dtα=1Γ(1−α)∫0t(t−u)−αf′(u)du

is the fractional Caputo time derivative, $f^{\prime }\left(u\right)=df\left(u\right)/du$.

Green’s function for Equation (3) is (see, among others, Refs. [29,30,31,32,33])

(5) $$\begin{array}{c} P_{\alpha }\left(x,t|x_{0}\right)=\frac{1}{2\sqrt{D_{\alpha }}}F_{-1+\alpha /2,\alpha /2}\left(t;\frac{|x-x_{0}|}{\sqrt{D_{\alpha }}}\right)=\frac{1}{2\sqrt{D_{\alpha }t^{\alpha }}}\sum_{j=0}^{\infty }\frac{1}{j!\Gamma (1-\alpha (j+1)/2)}{\left(-\frac{|x-x_{0}|}{\sqrt{D_{\alpha }t^{\alpha }}}\right)}^{j}. \end{array}$$

We mention that the solutions of the ordinary subdiffusion equation can often be expressed by the function $F_{\nu ,\beta }$; see [27,34] and the references cited therein. This is defined as follows:

(6) $$F_{\nu ,\beta }\left(t;a\right)=\frac{1}{t^{1+\nu }}\sum_{j=0}^{\infty }\frac{1}{\Gamma (-\nu -\beta j)}{\left(-\frac{a}{t^{\beta }}\right)}^{j}=\frac{1}{\beta a^{(1+\nu )/\beta }}H_{11}^{10}\left(\frac{a^{1/\beta }}{t}\left|\begin{array}{c} \left(1,1\right)\\ \left((1+\nu )/\beta ,1/\beta \right) \end{array}\right.\right),\;a,\beta >0,$$

where *H* denotes the Fox H-function; see [35]. The special case of Equation (6), namely the function $F_{-1+\alpha /2,\alpha /2}$, is also called the Mainardi function.

Formally, Green’s function for normal diffusion can be obtained from Green’s function for the ordinary subdiffusion equation, given in Equation (5), in the limit $\alpha \to 1^{-}$; in the following, this limit is also noted as α=1.

(7) $$P_{\alpha =1}\left(x,t|x_{0}\right)=\frac{1}{2\sqrt{\pi Dt}}\;e^{-\frac{{\left(x-x_{0}\right)}^{2}}{4Dt}}.$$

The above function fulfils the normal diffusion equation

(8) $$\frac{\partial P_{\alpha =1}\left(x,t|x_{0}\right)}{\partial t}=D\frac{\partial^{2}P_{\alpha =1}\left(x,t|x_{0}\right)}{\partial x^{2}}.$$

In terms of the ordinary Laplace transform,

(9) $$L\left[f\left(t\right)\right]\left(s\right)=\int_{0}^{\infty }e^{-st}f\left(t\right)dt,$$

due to the following relation:

(10) LdαCf(t)dtα(s)=sαL[f(t)](s)−sα−1f(0),α∈(0,1),

the ordinary subdiffusion equation is

(11) $$s^{\alpha }L\left[P_{\alpha }\left(x,t|x_{0}\right)\right]\left(s\right)-s^{\alpha -1}P_{\alpha }\left(x,0|x_{0}\right)=D_{\alpha }\frac{\partial^{2}L\left[P_{\alpha }\left(x,t|x_{0}\right)\right]\left(s\right)}{\partial x^{2}}.$$

The solution to Equation (11) is the Laplace transform of Green’s function:

(12) $$L\left[P_{\alpha }\left(x,t|x_{0}\right)\right]\left(s\right)=\frac{1}{2\sqrt{D_{\alpha }}s^{1-\alpha /2}}\;e^{-|x-x_{0}|\frac{s^{\alpha /2}}{\sqrt{D_{\alpha }}}}.$$

### 2.2. Factional Superdiffusion Equation

The fractional superdiffusion equation is

(13) $$\frac{\partial P_{\gamma }\left(x,t|x_{0}\right)}{\partial t}=D_{\gamma }\frac{\partial^{\gamma }P_{\gamma }\left(x,t|x_{0}\right)}{{\partial |x|}^{\gamma }},$$

where the Riesz-type fractional derivative is defined by its Fourier transform, $F\left(k\right)=\int_{-\infty }^{\infty }e^{ikx}f\left(x\right)dx$, as

(14) $$F\left[\frac{d^{\gamma }f\left(x\right)}{{d|x|}^{\gamma }}\right]\left(k\right)=-{\left|k\right|}^{\gamma }F\left(k\right).$$

Green’s function for Equation (13) is as follows (see Ref. [27] and the references cited therein):

(15) $$\begin{array}{cc} P_{\gamma }\left(x,t|x_{0}\right) & =\frac{1}{\sqrt{\pi }\left|x-x_{0}\right|}H_{12}^{11}\left(\frac{|x-x_{0}{|}^{\gamma }}{2^{\gamma }D_{\gamma }t}\left|\begin{array}{c} \left(1,1\right)\\ \left(1/2,\gamma /2)\;(1,\gamma /2\right) \end{array}\right.\right)\\ & =\frac{1}{\gamma \sqrt{\pi }{\left(D_{\gamma }t\right)}^{1/\gamma }}\sum_{j=0}^{\infty }\frac{\Gamma (1/\gamma +2j/\gamma )}{j!\Gamma (1/2+j)}{\left(-\frac{{\left(x-x_{0}\right)}^{2}}{4{\left(D_{\gamma }t\right)}^{2/\gamma }}\right)}^{j}. \end{array}$$

## 3. G-Subdiffusion Equation

The *g*-subdiffusion equation is a modified ordinary subdiffusion equation, as given in Equation (3). The modification consists of changing the time variable, *t*, to a function g(t),

(16) t→g(t),

where g(t) is given in units of time and meets the conditions

(17) $$g\left(0\right)=0,\;\;g\left(\infty \right)=\infty ,\;\;g^{\prime }\left(t\right)>0.$$

In order to determine the equation and Green’s function for the *g*-subdiffusion process, the Laplace transform with respect to the function *g*, which is called the *g*-Laplace transform, can be used [36,37]:

(18) $$L_{g}\left[f\left(t\right)\right]\left(s\right)=\int_{0}^{\infty }e^{-sg(t)}f\left(t\right)g^{\prime }\left(t\right)dt.$$

The relationship between the Laplace transforms is as follows:

(19) $$L_{g}\left[f\left(t\right)\right]\left(s\right)=L\left[f\left(g^{-1}\left(t\right)\right)\right]\left(s\right).$$

Equation (19) provides the relation

(20) $$L_{g}\left[f\left(t\right)\right]\left(s\right)=L\left[h\left(t\right)\right]\left(s\right)\Leftrightarrow f\left(t\right)=h\left(g\left(t\right)\right).$$

Knowing the ordinary Laplace transform, the above equation is helpful in determining the inverse *g*-Laplace transform. For example, since $L^{-1}\left[1/s^{\mu +1}\right]\left(t\right)=t^{\mu }/\Gamma \left(1+\mu \right)$, μ>−1, and $L^{-1}\left[s^{\nu }e^{-as^{\beta }}\right]\left(t\right)=F_{\nu ,\beta }\left(t;a\right)$, a,β>0 [29], we obtain

(21) $$L_{g}^{-1}\left[\frac{1}{s^{\mu +1}}\right]\left(t\right)=\frac{g^{\mu }\left(t\right)}{\Gamma (1+\mu )},\;\mu >-1,$$

(22) $$\begin{array}{c} L_{g}^{-1}\left[s^{\nu }e^{-as^{\beta }}\right]\left(t\right)\equiv F_{\nu ,\beta }\left(g\left(t\right);a\right)=\frac{1}{g^{1+\nu }\left(t\right)}\sum_{j=0}^{\infty }\frac{1}{j!\Gamma (-\nu -\beta j)}{\left(-\frac{a}{g^{\beta }\left(t\right)}\right)}^{j},\;\;a,\beta >0. \end{array}$$

From Equation (20), it can be concluded that the change in the time variable in the subdiffusion equation can be made using the relation

(23) $$t\to g\left(t\right)\Leftrightarrow L\left[P_{\alpha }\left(x,t|x_{0}\right)\right]\left(s\right)\to L_{g}\left[P_{g}\left(x,t|x_{0}\right)\right]\left(s\right).$$

By applying the rule given in Equation (23) to Equation (11), we obtain

(24) $$s^{\alpha }L_{g}\left[P_{g,\alpha }\left(x,t|x_{0}\right)\right]\left(s\right)-s^{\alpha -1}P_{g,\alpha }\left(x,0|x_{0}\right)=D_{\alpha }\frac{\partial^{2}L_{g}\left[P_{g,\alpha }\left(x,t|x_{0}\right)\right]\left(s\right)}{\partial x^{2}},$$

where $P_{g,\alpha }\left(x,0|x_{0}\right)=\delta \left(x-x_{0}\right)$. Due to the relation

(25) LgdgαCf(t)dtα(s)=sαLg[f(t)](s)−sα−1f(0),α∈(0,1),

where

(26) dgαCf(t)dtα=1Γ(1−α)∫0t[g(t)−g(u)]−αf′(u)du

is the Caputo fractional derivative with respect to another function *g* [21,36,37], the inverse *g*-Laplace transform of Equation (25) provides the *g*-subdiffusion equation:

(27) ∂gαCPg,α(x,t|x0)∂tα=Dα∂2Pg,α(x,t|x0)∂x2.

When $\alpha \to 1^{-}$, we obtain

(28) limα→1−dgαCf(t)dtα=f′(t)g′(t).

Combining Equations (12) and (23), we obtain Green’s function for *g*-subdiffusion equation in terms of the *g*-Laplace transform:

(29) $$L_{g}\left[P_{g,\alpha }\left(x,t|x_{0}\right)\right]\left(s\right)=\frac{1}{2\sqrt{D_{\alpha }}s^{1-\alpha /2}}\;e^{-|x-x_{0}|\frac{s^{\alpha /2}}{\sqrt{D_{\alpha }}}}.$$

Equations (22) and (29) provide the following equation:

(30) $$\begin{array}{c} P_{g,\alpha }\left(x,t|x_{0}\right)=\frac{1}{2\sqrt{D_{\alpha }}}f_{-1+\alpha /2,\alpha /2}\left(g\left(t\right);\frac{|x-x_{0}|}{\sqrt{D_{\alpha }}}\right)=\frac{1}{2\sqrt{D_{\alpha }g^{\alpha }\left(t\right)}}\sum_{j=0}^{\infty }\frac{1}{j!\Gamma (1-\alpha (j+1)/2)}{\left(-\frac{|x-x_{0}|}{\sqrt{D_{\alpha }g^{\alpha }\left(t\right)}}\right)}^{j}. \end{array}$$

Since *P* is translationally invariant and symmetric with respect to the point $x_{0}$, we obtain

(31) $$\sigma^{2}\left(t\right)=2\int_{0}^{\infty }x^{2}P\left(x,t|0\right)dx.$$

In terms of the *g*-Laplace transform, we have $L_{g}\left[\sigma^{2}\left(t\right)\right]\left(s\right)=2\int_{0}^{\infty }x^{2}L_{g}\left[P_{g,\alpha }\left(x,t|0\right)\right]\left(s\right)dx=2D_{\alpha }/s^{1+\alpha }$. Finally, for *g*-subdiffusion, we obtain

(32) $$\sigma_{g}^{2}\left(t\right)=\frac{2D_{\alpha }}{\Gamma (1+\alpha )}g^{\alpha }\left(t\right).$$

## 4. Using the G-Subdiffusion Equation to Describe Superdiffusion

The idea of using the *g*-subdiffusion equation to describe superdiffusion is based on the definition of the function *g*, which allows Equation (32) to be written in the form of Equation (2) with finite κ.

### 4.1. Finding the Function *g*

We find a function *g* which ensures that Green’s function for the *g*-subdiffusion equation given Equation (30) is asymptotically consistent with that obtained for fractional superdiffusion, i.e., Equation (15):

(33) $$P_{g,\alpha }\left(x,t\to \infty |x_{0}\right)=P_{\gamma }\left(x,t\to \infty |x_{0}\right).$$

Since

(34) $$P_{g,\alpha }\left(x,t\to \infty |x_{0}\right)=\frac{1}{2\sqrt{D_{\alpha }\Gamma \left(1-\alpha /2\right)g^{\alpha }\left(t\right)}},$$

(35) $$P_{\gamma }\left(x,t\to \infty |x_{0}\right)=\frac{1}{\gamma \sqrt{\Gamma (1/\gamma )}{\left(D_{\gamma }t\right)}^{1/\gamma }},$$

from the above equations, we obtain

(36) $$\tilde{g}\left(t\right)=Et^{\frac{2}{\gamma \alpha }},$$

where

(37) $$E={\left(\frac{\pi \gamma D_{\gamma }^{1/\gamma }}{2\sqrt{D_{\alpha }}\Gamma \left(1/\gamma \right)\Gamma \left(1-\alpha /2\right)}\right)}^{2/\alpha },$$

and $\tilde{g}$ represents the function *g* for superdiffusion. We note that $\sqrt{g^{\alpha }\left(t\right)D_{\alpha }}=\pi \gamma D_{\gamma }^{1/\gamma }t^{1/\gamma }/$$\left[2\Gamma \left(1/\gamma \right)\sqrt{\pi }\right]$, which causes the subdiffusion coefficient, $D_{\alpha }$, to be eliminated from Green’s function, $P_{\tilde{g},\alpha }$. Equations (30) and (36) provide Green’s function for describing superdiffusion:

(38) $$\begin{array}{c} P_{\tilde{g},\alpha }\left(x,t|x_{0}\right)=\frac{\Gamma (1/\gamma )\Gamma (1-\alpha /2)}{\pi \gamma {\left(D_{\gamma }t\right)}^{1/\gamma }}\sum_{j=0}^{\infty }\frac{1}{j!\Gamma (1-\alpha (j+1/2))}{\left(-\frac{|x-x_{0}|2\Gamma \left(1/\gamma \right)\Gamma \left(1-\alpha /2\right)}{\pi \gamma {\left(D_{\gamma }t\right)}^{1/\gamma }}\right)}^{j}. \end{array}$$

From Equations (32), (36), and (37), we obtain

(39) $$\sigma^{2}\left(t\right)=\kappa_{\tilde{g}}t^{\frac{2}{\gamma }},$$

with

(40) $$\kappa_{\tilde{g}}={\left(\frac{\pi \gamma D_{\gamma }^{1/\gamma }}{\sqrt{2}\Gamma \left(1/\gamma \right)\Gamma \left(1-\alpha /2\right)}\right)}^{2}.$$

### 4.2. *G*-Superdiffusion Equation

The *g*-superdiffusion equation, which is defined as the *g*-subdiffusion equation describing superdiffusion, is

(41) ∂γ,αCP(x,t|x0)∂tγ,α=D˜∂2P(x,t|x0)∂x2,

where

(42) ∂γ,αCf(t)∂tγ,α=1Γ(1−α)∫0tf′(u)(t2γα−u2γα)du,

and $\tilde{D}$ is the superdiffusion coefficient given in units of $m^{2}/\sec^{2/\gamma }$. This coefficient is related to other parameters as $\tilde{D}={\left[\pi \gamma D_{\gamma }^{1/\gamma }/\left(2\Gamma \left(1/\gamma \right)\Gamma \left(1-\alpha /2\right)\right)\right]}^{2}$.

### 4.3. Stochastic Interpretation

The *g*-subdiffusion equation can be derived from a modified continuous-time random walk (CTRW) model (called the *g*-CTRW model), which becomes the standard CTRW model when g(t)≡t [27,38]. The idea behind this model is as follows. Let $\Delta t_{i}$ be the waiting time for the particle to carry out its *i*-th jump. The sequences of waiting times for the particle to jump for both processes are related to each other as follows:

(43) $$P_{n}\left[\underset{ordinary\;subdif.}{\underset{︸}{\left(\Delta t_{1},\Delta t_{2},\ldots ,\Delta t_{n}\right)}}\right]=P_{n}\left[\underset{g\;subdif.}{\underset{︸}{\left(g^{-1}\left(\Delta t_{1}\right),g^{-1}\left(\Delta t_{2}\right),\ldots ,g^{-1}\left(\Delta t_{n}\right)\right)}}\right],$$

where $P_{n}$ is the probability distribution of a sequence of *n* jumps. The average number of particle jumps for *g*-subdiffusion is given by the formula [27]

(44) $$\langle n(t)\rangle =\frac{g^{\alpha }\left(t\right)}{\tau \Gamma (1+\alpha )},$$

where τ is a parameter given in units of $\sec^{\alpha }$. The mean jump frequency is defined as $f_{q}\left(t\right)=d\langle n(t)\rangle /dt$; for *g*-subdiffusion, this is

(45) $$f_{q}\left(t\right)=\frac{g^{\prime }\left(t\right)}{\tau \Gamma \left(\alpha \right)g^{1-\alpha }\left(t\right)}.$$

From Equations (36) and (45), we obtain

(46) $$f_{q}\left(t\right)=\tilde{E}t^{\frac{2}{\gamma }-1}.$$

where $\tilde{E}=2E^{\alpha }/\left(\gamma \tau \Gamma \left(1+\alpha \right)\right)$. Equation (46) shows that the superdiffusion effect in the *g*-subdiffusion process is caused by an increasing frequency of particle jumps. This is a different superdiffusion interpretation than its interpretation within the standard CTRW model, in which the superdiffusion effect originates from anomalously long particle jumps performed with relatively high probabilities, while the jump frequency is constant.

### 4.4. The Influence of Parameter α on *g*-Superdiffusion

**Figure 1:**
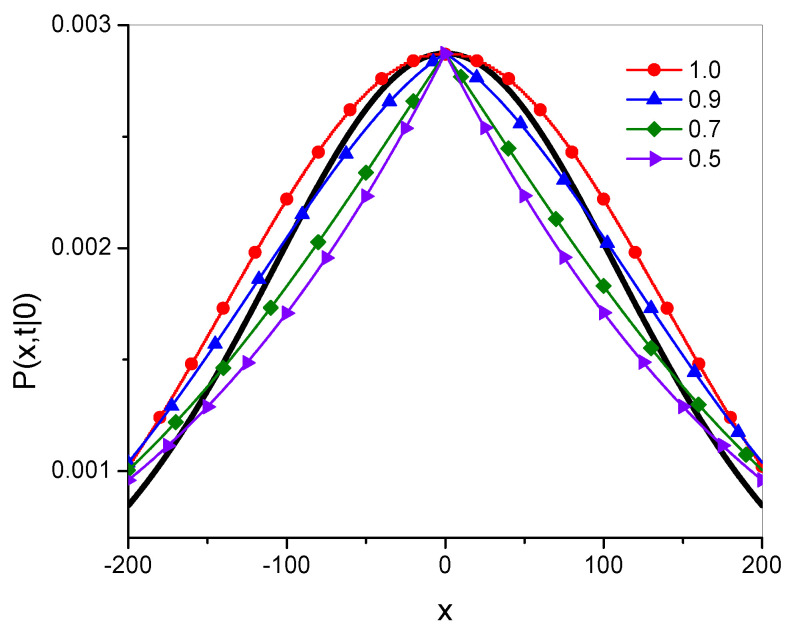
Green’s functions for fractional subdiffusion, $P_{\gamma }$, given in Equation (15) (thick solid lines without symbols), and for *g*-superdiffusion, $P_{\tilde{g},\alpha }$, given in Equation (38) (lines with symbols), for α given in the legend; here, t=100, $D_{\gamma }=10$, and $x_{0}=0$.

Example plots of the Green’s functions $P_{\gamma }$ and $P_{\tilde{g},\alpha }$ are shown in Figure 1; these Green’s functions were plotted for the 20 leading terms in the series defining the function. Throughout this paper, the values of all parameters and variables are given in arbitrarily chosen units.

The qualitative differences between the functions are most visible at the point x=0. The function $P_{\gamma }$ is smooth, as is the function $P_{\tilde{g},\alpha }$ for $\alpha \to 1^{-}$, while the latter function has characteristic spikes at this point for α<1.

We note that the exponent of the function in Equation (39) is the same as for fractional superdiffusion and depends on the superdiffusion parameter γ only. The function $\kappa_{\tilde{g}}$ is finite and depends on both parameters γ and α. In order to check the influence of the parameter α on Green’s function, we use the relative function $P_{R}$, showing the relative difference between the Green’s functions $P_{\tilde{g},\alpha }$ and $P_{\gamma }$:

(47) $$P_{R}\left(x,t|x_{0}\right)=\frac{P_{\gamma }\left(x,t|x_{0}\right)-P_{\tilde{g},\alpha }\left(x,t|x_{0}\right)}{P_{\gamma }\left(x,t|x_{0}\right)}.$$

**Figure 2:**
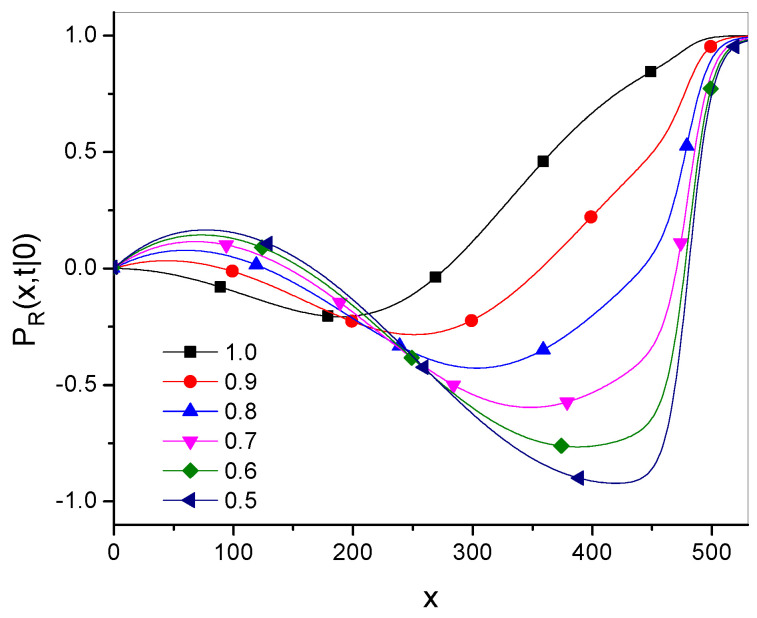
Plots of the relative function $P_{R}$ for α given in the legend, with γ=1.5, t=100, and $D_{\gamma }=10$.

An example of the influence of the parameter α on Green’s function is shown in Figure 2.

The figure suggests that for a value of *x* not too far from the initial particle position, the functions $P_{\gamma }$ and $P_{\tilde{g},\alpha }$ differ from each other a little, and $P_{\gamma }$ is closer to $P_{\tilde{g},\alpha }$ for larger values of α. For large values of *x*, $P_{\gamma }$ dominates over $P_{\tilde{g},\alpha }$.

### 4.5. *G*-Subdiffusion for $\alpha \to 1^{-}$

Let us write the function $P_{\gamma }$, given in Equation (15), in the following form:

(48) $$P_{\gamma }\left(x,t|x_{0}\right)=\frac{1}{\gamma \sqrt{\pi }{\left(D_{\gamma }t\right)}^{1/\gamma }}\sum_{j=0}^{\infty }\frac{A_{j}}{j!}{\left(-\frac{{\left(x-x_{0}\right)}^{2}}{4{\left(D_{\gamma }t\right)}^{2/\gamma }}\right)}^{j},$$

where $A_{j}=\Gamma \left(1/\gamma +2j/\gamma \right)/\left[\Gamma \left(1/2+j\right)\right]$. In the limit $\alpha \to 1^{-}$, $P_{\tilde{g},\alpha }$ has a structure similar to $P_{\gamma }$,

(49) $$P_{\tilde{g},\alpha \to 1^{-}}\left(x,t|x_{0}\right)=\frac{1}{\gamma \sqrt{\pi }{\left(D_{\gamma }t\right)}^{1/\gamma }}\sum_{j=0}^{\infty }\frac{B_{j}}{j!}{\left(-\frac{{\left(x-x_{0}\right)}^{2}}{4{\left(D_{\gamma }t\right)}^{2/\gamma }}\right)}^{j},$$

where $B_{j}=\left[\Gamma \left(1/\gamma \right)/\sqrt{\pi }\right]{\left[2\Gamma \left(1/\gamma \right)/\sqrt{\pi }\gamma \right]}^{2j}$.

**Figure 3:**
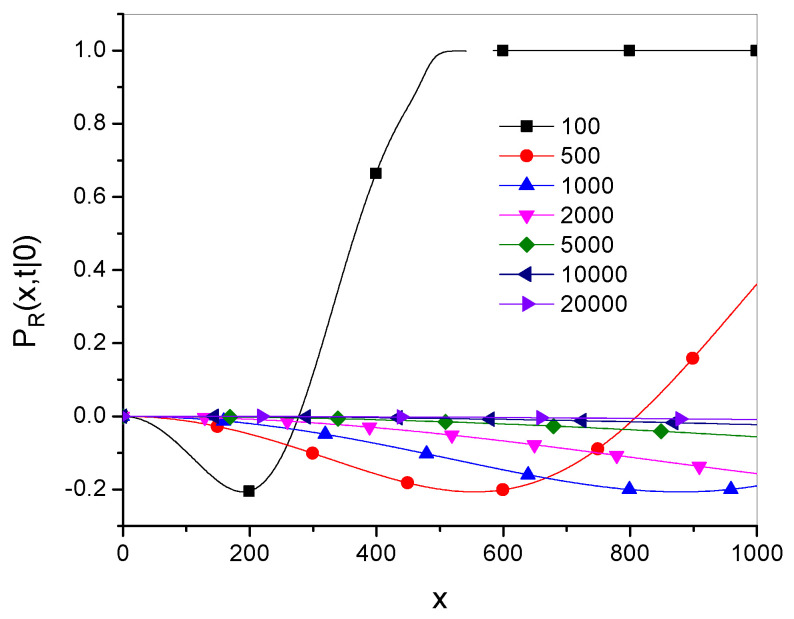
Plots of $P_{R}$, given in Equation (50), for the times given in the legend, with γ=1.5.

**Figure 4:**
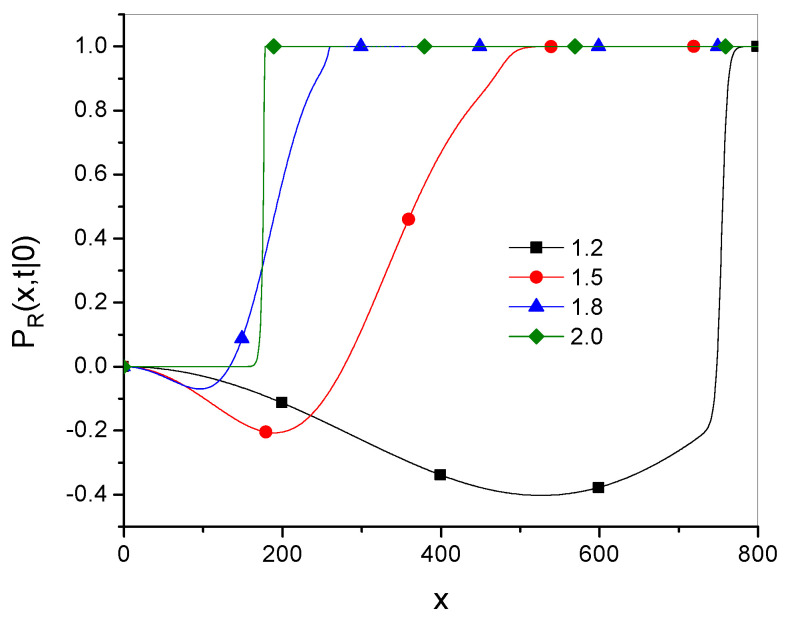
Plots of $P_{R}$, given in Equation (50), for γ given in the legend, with t=100.

Plots of the relative function

(50) $$P_{R}\left(x,t|x_{0}\right)=\frac{P_{\gamma }\left(x,t|x_{0}\right)-P_{\tilde{g},\alpha \to 1^{-}}\left(x,t|x_{0}\right)}{P_{\gamma }\left(x,t|x_{0}\right)}$$

are shown in Figure 3 and Figure 4, where $D_{\gamma }=10$ and $x_{0}=0$.

Figure 3 shows that the range of *x* in which both Green’s functions are close to each other grows over time. Figure 4 shows that for larger values of the parameter γ (which corresponds to a smaller superdiffusion effect), the relation $P_{\gamma }\gg P_{\tilde{g},\alpha \to 1^{-}}$ holds over a larger range of *x*.

**Figure 5:**
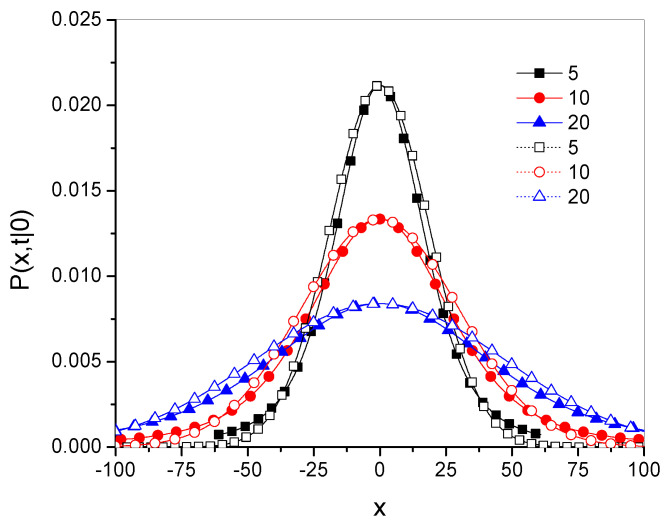
Green’s functions for fractional subdiffusion $P_{\gamma }$, given in Equation (15) (solid lines with filled symbols), and for *g*-superdiffusion $P_{\tilde{g},\alpha }$, given in Equation (38) (dashed lines with open symbols), for the times given in the legend.

**Figure 6:**
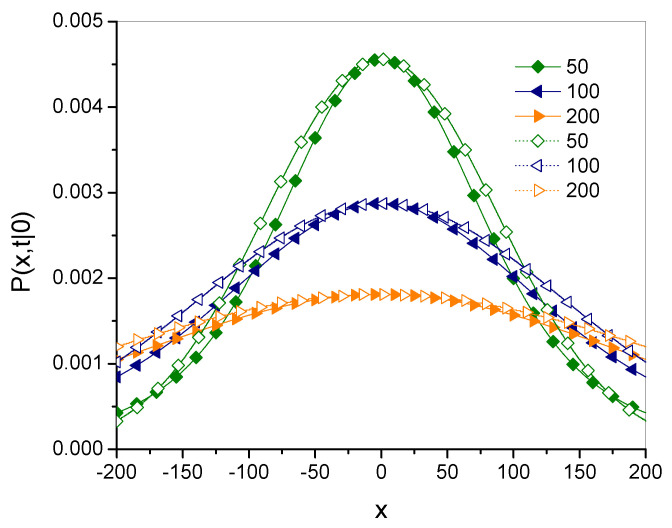
The plot description is the same as for Figure 5.

**Figure 7:**
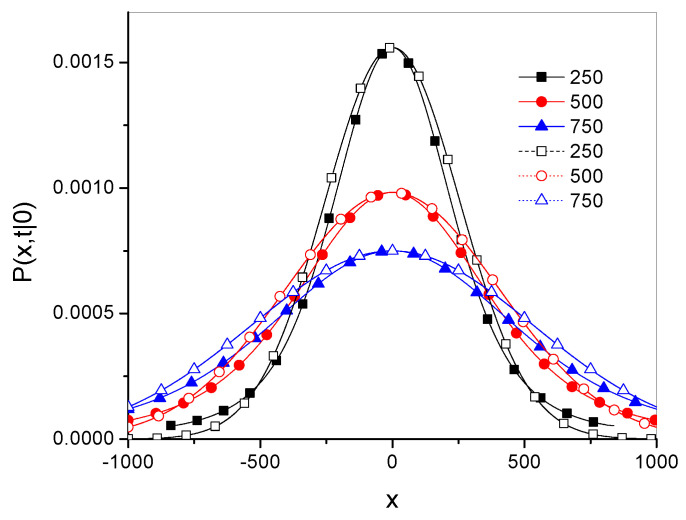
The plot description is the same as for Figure 5.

Plots of the Green’s functions $P_{\gamma }$ and $P_{\tilde{g},\alpha \to 1^{-}}$ for different times are shown in Figure 5, Figure 6 and Figure 7 for γ=1.5, $D_{\gamma }=10$, and $x_{0}=0$.

These plots suggest that both functions are rather close to each other for both short and long time ranges. Their qualitative features are also similar.

## 5. Filtration in a Superdiffusion System

As mentioned, using the fractional superdiffusion equation, one cannot uniquely define local boundary conditions at a thin membrane, excluding boundary conditions at fully absorbing or fully reflecting walls [39]. The boundary conditions used for this equation are usually nonlocal, which causes difficulties in their physical interpretation. However, for the *g*-superdiffusion equation, local boundary conditions can be used because the equation contains an integer-order spatial derivative; these conditions are, in practice, the same as the boundary conditions for ordinary subdiffusion or normal diffusion equations.

A membrane can be used to filter a diffusing substance. Assuming that the system is homogeneous in the plane parallel to the membrane, the problem is one-dimensional. Let a thin membrane, placed at the point x=0, separate vessels *A* and *B*. We assume that, initially, a diffusing molecule is in vessel *A*, with $x_{0}<0$. The filtering membrane allows for the (almost) free movement of molecules from *A* to *B*, while molecules trying to pass through the membrane in the opposite direction can be retained at the membrane with probability σ. Let us assume that the walls limiting the vessels are located at a large distance from the membrane and do not effectively affect the diffusion of molecules through the membrane. Then, the vessels are represented as infinite intervals, A=(−∞,0) and B=(0,∞).

The boundary conditions at the membrane are [34]

(51) $$J_{A,\tilde{g},\alpha }\left(0^{-},t|x_{0}\right)=J_{B,\tilde{g},\alpha }\left(0^{+},t|x_{0}\right)$$

and

(52) $$P_{A,\tilde{g},\alpha }\left(0^{-},t|x_{0}\right)=\sigma P_{B,\tilde{g},\alpha }\left(0^{+},t|x_{0}\right),$$

where the *g*-superdiffusion flux, $J_{\tilde{g},\alpha }$, is defined as

(53) Jg˜,α(x,s|x0)=−Dα∂g˜αC∂tα∂Pg˜,α(x,t|x0)∂x.

The above boundary conditions generate the following Green’s functions (see Ref. [34]):

(54) $$P_{A,\tilde{g},\alpha }\left(x,t|x_{0}\right)=P_{\tilde{g},\alpha }\left(x,t|x_{0}\right)+\left(1-\Lambda \right)P_{\tilde{g},\alpha }\left(x,t|-x_{0}\right),$$

(55) $$P_{B,\tilde{g},\alpha }\left(x,t|x_{0}\right)=\Lambda P_{\tilde{g},\alpha }\left(x,t|x_{0}\right),$$

where Λ=2σ/(1+σ).

As an example, we consider a filtration process taking place in a subdiffusive medium, such as a turbulent one, in which at the initial moment, a homogeneous solution of concentration $C_{0}$ is in region *A* and there is no diffusing substance in region *B*. The initial conditions are $C_{A}\left(x,0\right)=C_{0}$ and $C_{B}\left(x,0\right)=0$. We are interested in the temporal evolution of the amount of substance in region *B*. The concentration, $C_{B}\left(x,t\right)$, can be calculated using the formula

(56) $$C_{B,\tilde{g},\alpha }\left(x,t\right)=C_{0}\int_{-\infty }^{0}P_{B,\tilde{g},\alpha }\left(x,t|x_{0}\right)dx_{0}.$$

We obtain

(57) $$C_{B,\tilde{g},\alpha }\left(x,t\right)=\frac{\Lambda C_{0}}{2}F_{-1,\alpha /2}\left(Et^{\frac{2}{\gamma \alpha }},\frac{x}{\sqrt{D}}\right).$$

The evolution of the total amount of substance in region *B* over time, $W_{B,\tilde{g},\alpha }\left(t\right)=\int_{0}^{\infty }C_{B,\tilde{g},\alpha }\left(x,t\right)dx$, is

(58) $$W_{B,\tilde{g},\alpha }\left(t\right)=\frac{\Lambda C_{0}E^{\alpha /2}}{\Gamma (1+\alpha /2)}t^{\frac{1}{\gamma }}.$$

Equations (57) and (58) can easily be derived when we use the *g*-Laplace transform of the above equations and Equations (21), (22), (29), and (36). We add that for ordinary subdiffusion with the parameter α, the rate of the filtration process is $W_{B,\alpha }\left(t\right)∼t^{\alpha /2}$ [34]. By comparing this equation with Equation (58), we obtain the relation $W_{B,\tilde{g},\alpha }\left(t\right)∼t^{\eta }W_{B,\alpha }\left(t\right)$, with η=(1/γ)−(α/2)>0.

## 6. Final Remarks

The *g*-subdiffusion equation with the fractional Caputo derivative with respect to another function can be interpreted as the ordinary subdiffusion equation with a changed time variable. So far, the *g*-subdiffusion equation has mainly been used to describe a smooth transition from subdiffusion to another type of diffusion [20,27] or subdiffusion with a changed α parameter [28]. In this paper, this equation was used to describe superdiffusion over the entire time domain. The characteristic features of the *g*-superdiffusion equation are as follows:

- The *g*-superdiffusion equation is defined as the *g*-subdiffusion equation given in Equation (27) with the function *g* given by Equation (36). This equation can be written in an equivalent form as Equation (41), which contains a Caputo-type fractional time derivative controlled by two parameters, γ∈(1,2) and α∈(0,1). The parameter γ controls the exponent of the time evolution of the MSD given in Equation (39), which defines the type of diffusion. This parameter also defines the order of the Riesz-type derivative with respect to the spatial variable in the fractional superdiffusion equation, which gives the same Green’s function as the *g*-subdiffusion equation in the limit t→∞. The parameter α controls the rate of convergence of Green’s functions.
- More generally, due to the relation $C\left(x,t\right)=\int_{-\infty }^{\infty }C\left(x_{0},0\right)P\left(x,t|x_{0}\right)dx_{0}$, the solution to the *g*-subdiffusion equation asymptotically converges to the solution of the fractional superdiffusion equation when the initial conditions and the parameter γ are the same for both equations.
- It appears that the parameter α for which the Green’s functions for *g*-superdiffusion are qualitatively most similar to that for fractional superdiffusion is α=1. This case is considered in Section 4.5.
- The *g*-subdiffusion equation is “local in space”, so “typical” boundary conditions at partially permeable walls can be used in the superdiffusion model.
- The stochastic interpretation of the *g*-superdiffusion process is that the jump frequency of a diffusing particle increases over time to infinity. The probability distribution of the jump lengths of a diffusing molecule has finite moments.
- Green’s function for *g*-subdiffusion gives $\sigma^{2}\left(t\right)=\kappa t^{2/\gamma }$ with κ<∞.

An effective method for solving the *g*-superdiffusion equations is applying a Laplace transform with respect to the function $\tilde{g}$, as given in Equation (36).

## Data Availability

The original contributions presented in this study are included in the article. Further inquiries can be directed to the corresponding author.

## References

[1] Metzler R., Klafter J. (2000). The random walk’s guide to anomalous diffusion: A fractional dynamics approach. Phys. Rep..

[2] Metzler R., Klafter J., Sokolov I.M. (1998). Anomalous transport in external fields: Continuous time random walks and fractional diffusion equations extended. Phys. Rev. E.

[3] Compte A. (1996). Stochastic foundations of fractional dynamics. Phys. Rev. E.

[4] Denisov S.I., Kantz H. (2011). Continuous-time random walk with a superheavy-tailed distribution of waiting times. Phys. Rev. E.

[5] Montroll E.W., Weiss G.H. (1965). Random walks on lattices. II. J. Math. Phys..

[6] Klafter J., Sokolov I.M. (2011). First Step in Random Walks. From Tools to Applications.

[7] Barkai E., Metzler R., Klafter J. (2000). From continuous time random walks to the fractional Fokker-Planck equation. Phys. Rev. E.

[8] Barkai E. (2001). Fractional Fokker-Planck equation, solution, and application. Phys. Rev. E.

[9] Klages R., Radons G., Sokolov I.M. (2008). Anomalous Transport: Foundations and Applications.

[10] Sokolov I.M., Klafter J., Blumen A. (2002). Fractional kinetics. Phys. Today.

[11] Sokolov I.M., Klafter J. (2005). From diffusion to anomalous diffusion: A century after Einstein’s Brownian motion. Chaos.

[12] Hilfer R., Anton L. (1995). Fractional master equations and fractal time random walks. Phys. Rev. E.

[13] Wyss W. (1986). The fractional diffusion equation. J. Math. Phys..

[14] Chechkin A.V., Klafter J., Sokolov I.M. (2003). Fractional Fokker-Planck equation for ultraslow kinetics. Europhys. Lett..

[15] Chechkin A.V., Gonchar V.Y., Gorenflo R., Korabel N., Sokolov I.M. (2008). Generalized fractional diffusion equations for accelerating subdiffusion and truncated Levy flights. Phys Rev. E.

[16] Barkai E., Garini Y., Metzler R. (2012). Strange kinetics of single molecules in living cells. Phys. Today.

[17] Metzler R., Jeon J.H., Cherstvy A.G., Barkai E. (2014). Anomalous diffusion models and their properties: Non-stationarity, non-ergodicity, and ageing at the centenary of single particle tracking. Phys. Chem. Chem. Phys..

[18] Cherstvy A.G., Safdari H., Metzler R. (2021). Anomalous diffusion, nonergodicity, and ageing for exponentially and logarithmically time–dependent diffusivity: Striking differences for massive versus massless particles. J. Phys. D Appl. Phys..

[19] Metzler R., Klafter J. (2004). The restaurant at the end of the random walk: Recent developments in the description of anomalous transport by fractional dynamics. J. Phys. A.

[20] Kosztołowicz T., Dutkiewicz A. (2021). Subdiffusion equation with Caputo fractional derivative with respect to another function. Phys. Rev. E.

[21] Almeida R. (2017). A Caputo fractional derivative of a function with respect to another function. Commun. Nonlinear Sci. Numer. Simul..

[22] Sokolov I.M. (2001). Thermodynamics and fractional Fokker-Planck equations. Phys. Rev. E.

[23] Feller W. (1968). An Introduction to Probability Theory and Its Applications, Volume 2.

[24] Chechkin A.V., Seno F., Metzler R., Sokolov I.M. (2017). Brownian yet non-Gaussian diffusion: From superstatistics to subordination of diffusing diffusivities. Phys. Rev. X.

[25] Dybiec B., Gudowska-Nowak E. (2010). Subordinated diffusion and continuous time random walk asymptotics. Chaos.

[26] Chechkin A., Sokolov I.M. (2021). Relation between generalized diffusion equations and subordination schemes. Phys. Rev. E.

[27] Kosztołowicz T. (2023). Subdiffusion equation with fractional Caputo time derivative with respect to another function in modeling transition from ordinary subdiffusion to superdiffusion. Phys. Rev. E.

[28] Kosztołowicz T., Dutkiewicz A. (2022). Composite subdiffusion equation that describes transient subdiffusion. Phys. Rev. E.

[29] Kosztołowicz T. (2004). From the solutions of diffusion equation to the solutions of subdiffusive one. J. Phys. A Math. Gen..

[30] Mainardi F. (1996). The fundamental solutions for the fractional diffusion–wave equation. Appl. Math. Lett..

[31] Mainardi F., Luchko Y., Pagnini G. (2001). The fundamental solutions of the space–time fractional diffusion equation. Fract. Calc. Appl. Anal..

[32] Mainardi F., Pagnini G., Saxena R.K. (2005). Fox H functions in fractional diffusion. J. Comput. Appl. Math..

[33] Apelblat A., Mainardi F. (2021). Application of the Efros theorem to the function represented by the inverse Laplace transform of *s*^−*μ*^e^−*sν*^. Symmetry.

[34] Kosztołowicz T. (2019). Model of anomalous diffusion-absorption process in a system consisting of two different media separated by a thin membrane. Phys. Rev. E.

[35] Mathai A.M., Saxena R.K., Haubold H.J. (2010). The H-Function. Theory and Applications.

[36] Fahad H.M., Rehman M.U., Fernandez A. (2021). On Laplace transforms with respect to functions and their applications to fractional differential equations. arXiv.

[37] Jarad F., Abdeljawad T. (2020). Generalized fractional derivatives and Laplace transform. Discret. Contin. Dyn. Syst.-Ser. S.

[38] Kosztołowicz T., Dutkiewicz A. (2021). Stochastic interpretation of *g*-subdiffusion process. Phys. Rev. E.

[39] Metzler R., Klafter J. (2000). Boundary value problems for fractional diffusion equations. Physica A.

